# Molecular and Evolutionary Characteristics of Chicken Parvovirus (ChPV) Genomes Detected in Chickens with Runting–Stunting Syndrome

**DOI:** 10.3390/v16091389

**Published:** 2024-08-30

**Authors:** Ruy D. Chacón, Christian J. Sánchez-Llatas, Antonio Charlys da Costa, Stefhany Valdeiglesias Ichillumpa, Pablo Cea-Callejo, Obert Marín-Sánchez, Claudete S. Astolfi-Ferreira, Silvana Santander-Parra, Luis F. N. Nuñez, Antonio J. Piantino Ferreira

**Affiliations:** 1 Department of Pathology, School of Veterinary Medicine, University of São Paulo, São Paulo 05508-270, Brazil; ruychaconv@alumni.usp.br (R.D.C.); csastolfi@gmail.com (C.S.A.-F.); ajpferr@usp.br (A.J.P.F.); 2 Department of Genetics, Physiology, and Microbiology, Faculty of Biology, Complutense University of Madrid, 28040 Madrid, Spain; chsanc01@ucm.es (C.J.S.-L.); pcea@ucm.es (P.C.-C.); 3 Laboratory of Virology (LIM 52), Department of Infectious Diseases, Instituto de Medicina Tropical, Faculdade de Medicina da Universidade de São Paulo, São Paulo 05403-000, Brazil; charlysbr@yahoo.com.br; 4 Laboratorio de Fisiología Molecular, Instituto de Investigación en Ganadería y Biotecnología, Facultad de Ingeniería Zootecnista, Agronegocios y Biotecnología, Universidad Nacional Toribio Rodríguez de Mendoza de Amazonas, Chachapoyas 01001, Peru; stefhanyvaldeiglesias@gmail.com; 5 Departamento Académico de Microbiología Médica, Facultad de Medicina, Universidad Nacional Mayor de San Marcos, Lima 15081, Peru; omarins@unmsm.edu.pe; 6 Facultad de Ciencias de la Salud, Carrera de Medicina Veterinaria, Universidad de Las Américas, Quito EC 170124, Ecuador; silvanahsp@yahoo.com; 7 One Health Research Group, Universidad de Las Américas, Quito EC 170124, Ecuador

**Keywords:** *Aveparvovirus*, runting–stunting syndrome, viral metagenomics, phylogenetic analysis, selective pressure, protein modeling, epitope prediction

## Abstract

Chicken Parvovirus (ChPV) belongs to the genus *Aveparvovirus* and is implicated in enteric diseases like runting–stunting syndrome (RSS) in poultry. In RSS, chicken health is affected by diarrhea, depression, and increased mortality, causing significant economic losses in the poultry industry. This study aimed to characterize the ChPV genomes detected in chickens with RSS through a metagenomic approach and compare the molecular and evolutionary characteristics within the *Aveparvovirus galliform1* species. The intestinal content of broiler flocks affected with RSS was submitted to viral metagenomics. The assembled prevalent genomes were identified as ChPV after sequence and phylogenetic analysis, which consistently clustered separately from Turkey Parvovirus (TuPV). The strain USP-574-A presented signs of genomic recombination. The selective pressure analysis indicated that most of the coding genes in *A. galliform1* are evolving under diversifying (negative) selection. Protein modeling of ChPV and TuPV viral capsids identified high conservancy over the VP2 region. The prediction of epitopes identified several co-localized antigenic peptides from ChPV and TuPV, especially for T-cell epitopes, highlighting the immunological significance of these sites. However, most of these peptides presented host-specific variability, obeying an adaptive scenario. The results of this study show the evolutionary path of ChPV and TuPV, which are influenced by diversifying events such as genomic recombination and selective pressure, as well as by adaptation processes, and their subsequent immunological impact.

## 1. Introduction

The genus *Aveparvovirus* belongs to the family *Parvoviridae*, subfamily *Parvovirinae*. According to the latest release from the International Committee on Taxonomy of Viruses (ICTV), it includes four species: *A. columbi1*, *A. gruiform1*, *A. passeriform1*, and *A. galliform1* [1]. *Aveparvovirus galliform1* species comprises the two viruses that infect domestic birds, Chicken Parvovirus (ChPV), and Turkey Parvovirus (TuPV).

Parvoviruses are small, icosahedral-shaped, non-enveloped viruses with a diameter of 19–24 nm and possess a single-stranded linear DNA genome. ChPV has a single-stranded genome with a length of approximately 5 kb. Its genome is flanked by two inverted terminal repeats (ITRs) and a coding region that includes the nonstructural protein (NS1), the overlapping structural viral capsid proteins VP1 and VP2, and the auxiliary proteins NP1 and NP [2,3,4]. The NS1 gene is conserved and is primarily used for *Aveparvovirus* species determination and molecular detection by PCR and qPCR [2,5]. On the other hand, the VP1 gene is responsible for the infectivity of the virus and the specificity of the host determinant between chickens and turkeys, and it is also used for molecular genotyping and the characterization of ChPV and TuPV [3,6].

Runting–stunting syndrome (RSS) is one of the most important enteric diseases in chicken and turkey health [7,8]. RSS has many synonyms, such as infectious stunting syndrome, pale-bird syndrome, malabsorption syndrome, malassimilation, and helicopter disease, and affects birds of all ages, from one-day-old chicks to mature hens [7,8,9]. This syndrome is composed of two aspects: runting and stunting. Runting refers to the reduced growth rate and size of the affected birds falling short of the expected weight. Stunting refers to the underdevelopment of various organs and systems in the bird’s body. This includes the gastrointestinal tract, immune system, and skeletal system. Stunted birds exhibit malformations, delayed maturation, and reduced function in these areas. In RSS, these conditions come together, resulting in economic losses for poultry producers as affected birds are less valuable and less productive. The principal externally observed signs in affected birds by RSS include apathy, depression, ruffled feathers, cloacal pasting, and diarrhea [7]. Regarding the etiology, RSS has been primarily associated with viruses, and many of them have been detected in chickens exhibiting signs of enteric disease resembling RSS [10,11].

ChPV and TuPV are associated with enteric diseases such as poult enteritis complex (PEC), poult enteritis mortality syndrome (PEMS) in turkeys, and runting–stunting syndrome (RSS) in broiler chickens [12]. ChPV primarily affects young chickens [13,14]. However, there have been many reports of ChPV infection in chickens of all ages [3,9,15]. The role of ChPV in RSS is still not well understood, primarily due to the difficulty of isolation [16]. Experimental infection with the ABU-P1 strain has successfully reproduced RSS, but no microscopic alterations were observed in the infected animals [14]. More studies need to be performed to elucidate this syndrome [12].

ChPV has been detected in chicken flocks of several countries, in both healthy-appearing birds and birds exhibiting signs of enteric disease, from one-day-old chicks to birds several weeks old [3,9,11,16,17]. In addition, novel molecular approaches such as genomics and metagenomics have been implemented to study and compare the role of viruses in enteric diseases and RSS, with a frequent identification of ChPV [18,19,20,21].

This study aimed to characterize the ChPV genomes detected in chickens with RSS through a metagenomic approach and compare the molecular and evolutionary characteristics within the *Aveparvovirus galliform1* species.

## 2. Materials and Methods

### 2.1. Clinical Samples and Viral Metagenomics

In this study, an analysis of intestinal content from a previously documented RSS investigation [15] was conducted. Sixteen pooled samples were subjected to viral metagenomics protocols as described elsewhere [22,23].

Briefly, a 50 mg aliquot of the pooled sample was diluted in HBSS and transferred to a tube containing lysing matrix C. The sample was homogenized and then centrifuged at 12,000× *g* for 10 min. The supernatant was filtered through a 0.45 μm filter to remove eukaryotic and bacterial debris.

To precipitate viral particles, cold PEG-it Virus Precipitation Solution was added to the filtrate, mixed gently, and incubated at 4 °C for 24 h. The mixture was then centrifuged at 10,000× *g* for 30 min at 4 °C. The supernatant was discarded, and the viral pellet was digested with a combination of nuclease enzymes at 37 °C for 2 h. Viral nucleic acids were then extracted using a ZR and ZR-96 Viral DNA/RNA Kit.

cDNA synthesis was performed using AMV reverse transcriptase (Promega, Madison, WI, USA). The second strand was synthesized with DNA Polymerase I Large (Klenow) Fragment (Promega, Madison, WI, USA). A DNA library was constructed using a Nextera XT Sample Preparation Kit (Illumina, San Diego, CA, USA) and labeled with dual barcodes. Pippin Prep (Sage Science, Beverly, MA, USA) was used for size selection (200–400 bp) with a target of 300 bp fragments. Deep sequencing was performed using Illumina sequencing (MiSeq 2 × 250 bases) with a Nextera™ XT Sample Preparation Kit (Illumina, San Diego, CA, USA).

Genomic assembly was followed by bioinformatics analysis following the strategy of Deng et al. [24]. These sequences were searched using BLASTx to identify similarities with viral proteins in the GenBank database. They were further compared against the GenBank non-redundant databases using BLASTn and BLASTx. After viral identification, full-length genomes were mapped against a reference sequence using Geneious R9 software (Biomatters Ltd. L2, 18 Shortland Street Auckland, 1010, New Zealand).

### 2.2. Sequence and Phylogenetic Analysis

Sequence and phylogenetic analyses focused on the NS and VP1 genes. This analysis included 74 complete coding genomes of *Aveparvoviruses* retrieved from GenBank: 71 *Aveparvovirus galliform1* strains (including two from this study) and 3 additional reference sequences from *Aveparvovirus gruiform1* and *Aveparvovirus passeriform1* species used as outgroups. Sequence alignment was performed using the online MAFFT service [25]. The optimal substitution model for the phylogenetic analysis was selected using ModelTest-NG v0.1.7 [26] based on the Bayesian Information Criterion (BIC). The maximum likelihood (ML) method was employed for tree reconstruction using PhyML [27]. Nodal support values were estimated through 1000 bootstrap replicates. The resulting phylogenetic tree was then visualized using iTOL [28]. Finally, sequence identity comparisons for the complete NS1 and VP1 genes were conducted using an identity matrix generated in Geneious R9 software.

### 2.3. Recombination Analysis

Screening for recombination signals and breakpoints was explored in 71 complete genomes of *Aveparvovirus galliform 1* strains using the Genetic Algorithm for Recombination Detection (GARD) [29].

The complete genomes of the two *Aveparvovirus galliform1* strains isolated in this study were analyzed to identify potential recombination events using RDP5 (version 4.97) as described by Martin et al. [30]. The employed methods included RDP, GENECONV, BootScan, MaxChi, Chimaera, SiScan, and 3Seq. Any potential recombination event was considered valid only if detected by five or more of these methods and had a *p*-value below 5 × 10^−4^.

### 2.4. Selective Pressure Analysis

To estimate the selective pressure on the entire gene as a unit, the Z test implemented in Mega11 using the Kumar model (K2P) was used [31]. Additionally, the Branch-site Unrestricted Statistical Test for Episodic Diversification (BUSTED) was used to detect positive selection on the entire gene if it had undergone positive selection on at least one site within at least one branch of the evolutionary tree [32]. The significance level for both methods was set to *p* ≤ 0.05.

Furthermore, we analyzed site-specific selection pressure. Episodic diversifying selection, where positive selection acts on a limited proportion of sites throughout the phylogeny, was investigated using mixed effects models of evolution (MEME). Detecting pervasive diversifying selection, which signifies sites consistently influenced by positive selection across the evolutionary tree, was performed through fixed-effects likelihood (FEL), fast unconstrained Bayesian approximation for inferring selection (FUBAR), and single-likelihood ancestor counting (SLAC) methods. Analyses were performed via the Datamonkey web server [32]. The significance level was set at *p* < 0.1 for MEME, FEL, and SLAC, while the FUBAR method used a posterior probability threshold of 0.9.

### 2.5. Protein Modeling of Viral Capsid and B and T Epitope Prediction

Protein modeling was performed to compare the viral capsid proteins VP1 (minor capsid protein) and VP2 (major capsid protein) as monomers, dimers, and trimers of ChPV and TuPV, using the consensus amino acid sequences for both viruses. VP2 overlaps with VP1 and is located 143 amino acids downstream. The protein structures were constructed using AlphaFold 3 [33]. The structures were then visualized and aligned using PyMOL [34]. The results were compared using the UniProt database (ChPV: ID D3X6W9, TuPV: ID D3X6X7).

T-cell linear epitopes were predicted for MHC class I using NetMHCpan 4.1 and for MHC class II using NetMHCIIpan 4.1 [35]. As proposed in a previous study [36], the best human substitute alleles for the chicken MHC were chosen for epitope prediction: three alleles for MHC class I (HLA*B 40:06, HLA*B 41:04, and HLA*B 41:03) and four DRB1 alleles for MHC class II (DRB1:1482, DRB1:1366, DRB1:1310, and DRB1:1445).

## 3. Results

### 3.1. Viral Metagenomics

Viral nucleic acids were enriched, amplified, and sequenced using Illumina MiSeq. Following the assembly and filtering of viral sequences, 33,160 viral reads were obtained. BLASTx analysis with an e-value threshold of 1 × 10^−5^ identified 37 distinct viral species (details in Table S1). The five most prominent families were *Parvoviridae* (26.80%), *Mimiviridae* (30.07%), *Pithoviridae* (9.48%), *Retroviridae* (18.63%), and *Siphoviridae* (7.19%). The most frequent genera included *Aveparvovirus* (25.16%), *Hokovirus* (21.57%), *Alpharetrovirus* (9.15%), *Pandoravirus* (1.63%), and *Protoparvovirus* (1.63%). The five most prevalent species were *Aveparvovirus galliform1* (25.16%), *Hokovirus* HKV1 (21.57%), *Pithovirus* LCPAC101 (8.50%), *Avian leukosis virus* (7.19%), and *Avian endogenous retrovirus* EAV-HP (6.54%).

Two near-complete *Aveparvovirus galliform1* genomes were assembled from the filtered reads and deposited in GenBank with accession numbers PP329606 (USP-574-A) and PP329607 (USP-574-B).

### 3.2. Sequence and Phylogenetic Analysis

**Figure 1:**
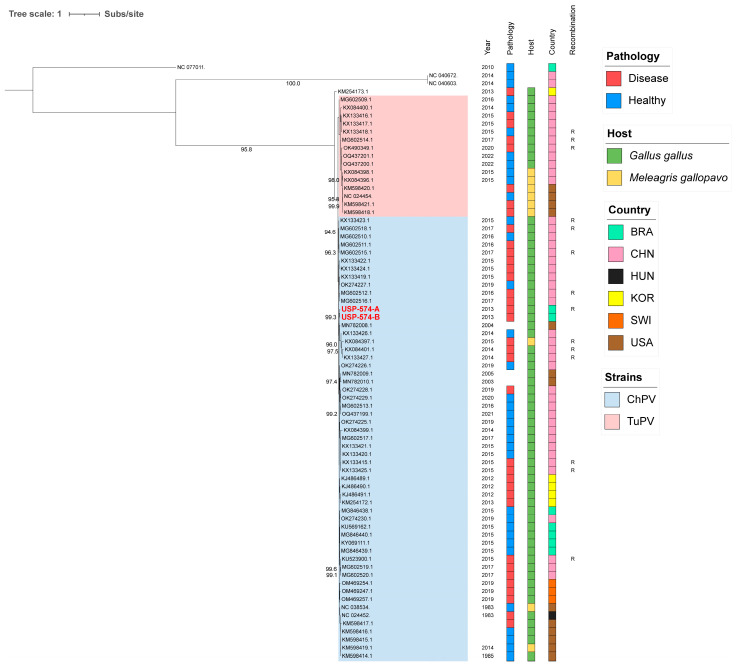
Phylogenetic analysis of 74 NS1 complete gene sequences of *Aveparvovirus*. The phylogenetic tree was inferred using PhyML through maximum-likelihood analysis under the HKY + R4 nucleotide substitution model with nodal support values based on 1000 bootstrap replicates. The color codes for the Pathology, Host, and Country are represented in vertical bars on the right of the names and in the legend. *Aveparvovirus* clades are colored and indicated in the legend. Brazilian strains USP-574-A and USP-574-B of this study were highlighted in red.

**Figure 2:**
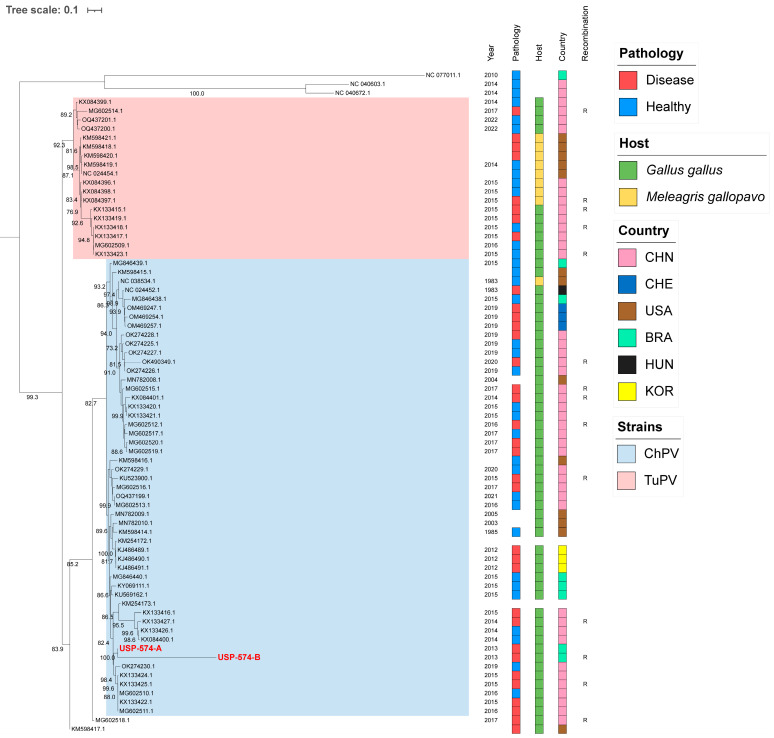
Phylogenetic analysis of 74 VP1 complete gene sequences of *Aveparvovirus*. The phylogenetic tree was inferred using PhyML through maximum-likelihood analysis under the GTR + R nucleotide substitution model with nodal support values based on 1000 bootstrap replicates. The color codes for the Pathology, Host, and Country are represented in vertical bars on the right of the names and in the legend. *Aveparvovirus* clades are colored and indicated in the legend. Brazilian strains USP-574-A and USP-574-B of this study were highlighted in red.

For these analyses, the two major *Aveparvovirus* genes were used: NS1 and VP1. The phylogenetic analysis of the NS1 gene permitted the distinguishing of two clades, including all sequences belonging to the *Aveparvovirus galliform1* (Figure 1). One of them included strains mostly isolated from chickens (Clade ChPV), while the other one included almost all strains isolated from turkeys together with others isolated from chickens (Clade TuPV). Brazilian strains of this study presented nucleotide identities of 98.5% between them, from 90.0% to 96.6% against clade ChPV and from 88.1% to 92.4% against clade TuPV (Table S2). Phylogenetic analyses of the VP1 gene differentiated the sequences of TuPV and those of ChPV, except for 10 ChPV sequences that are phylogenetically closer to TuPV (Figure 2). In turn, the ChPV clade included three subclades defined as ChPV-1, ChPV-2, and ChPV-3. The two sequences USP-574-A and USP-574-B presented in this study were included in different subclades (ChPV-2 and ChPV-3). Brazilian strains of this study presented nucleotide identities of 94.8% between them, from 80.7% to 96.0% against clade ChPV and from 71.9% to 79.9% against clade TuPV (Table S3). In all phylogenetic analyses, clustering by a year of isolation, country, or pathology was not observed.

### 3.3. Recombination Analysis

**Figure 3:**
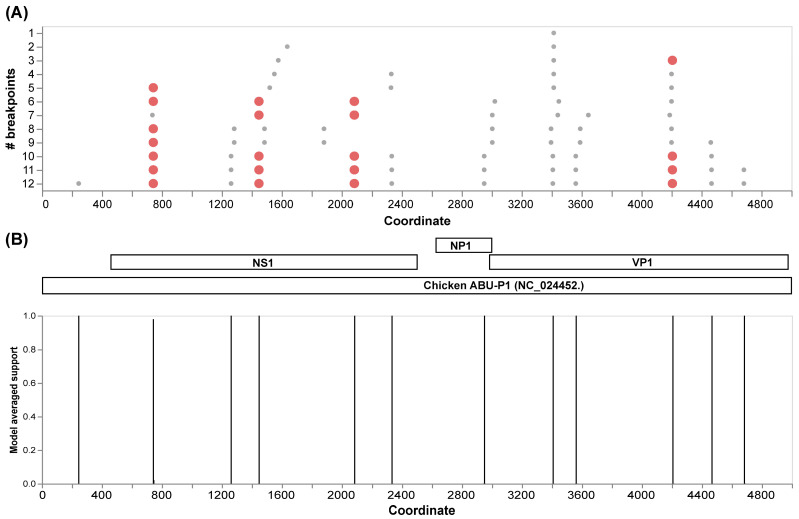
Recombination analysis of 71 VP1 complete genome sequences of *Aveparvovirus galliform1* performed with GARD. (**A**) Identification of inferred breakpoints along the multiple genome sequence alignment. Orange circles represent the best place of inferred breakpoints. (**B**) Localization of the inferred breakpoints along the complete genome and coding genes of Chicken Parvovirus reference genome ABU-P1 (NC_024452).

The initial recombination analysis performed with GARD allowed us to identify 12 inferred breakpoints along the alignment of the complete genomes (Figure 3A). Among these, four presented the best signals to be considered as recombination hotspots in *Aveparvovirus galliform1*. Three of these sites are located in the NS1 gene and one in the VP1/VP2 gene (Figure 3B).

**Figure 4:**
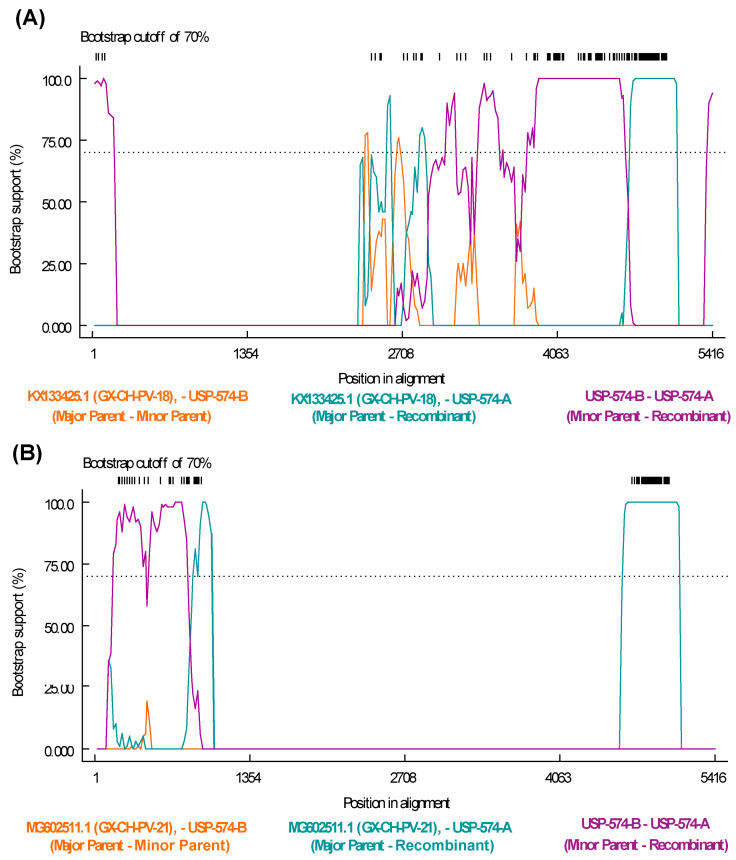
Recombination events detected in *Aveparvovirus galliform1* strain USP-574-A. (**A**) Bootscan analysis of event 50 was performed with GX-CH-PV-21 (MG602511) strain as the major parent and USP-574-B (PP329607) as the minor parent. (**B**) Bootscan analysis of event 50 was performed with GX-CH-PV-18 (KX133425) strain as the major parent and USP-574-B (PP329607) as the minor parent. Both images consider the vertical axis as the percentage of permuted trees and the horizontal axis as the position on the polyprotein of the query sequence.

The recombination analysis to identify potential recombinant strains permitted to identify 77 recombination events among all the sequences included in the analysis (Table S4). Specifically, potential recombination events explored for Brazilian strains from this study indicate that the strain USP-574-A underwent different recombination processes, with two potential recombination events supported by six and seven statistical methods, respectively (Table S4). The recombination regions were identified at positions 243–840 of the NS gene and 2277–3903 of the VP1 gene (Figure 4).

### 3.4. Selective Pressure Analysis

Initial analyses were performed on the entire genes as units to determine if these genes were under positive or negative selection. The codon-based Z test estimated that genes NS1, NP1, and VP1 evolved under purifying (negative) selection (dS > dN, *p*-value = 0.00), with (dN − dS) values of −15.27, −4.07, and −13.41, respectively. In contrast, the dN−dS value for gene NP was 0.38, suggesting potential evolution under positive selection, although lacking significant *p*-value support (*p*-value = 0.36). On the other hand, BUSTED analyses indicated that at least one site in NS1, NP, and VP1 experienced diversifying selection events (Table 1).

Next, we performed an analysis of the individual sites of the complete genes to determine the presence of episodic positive selection events (through MEME) and pervasive positive selection events (through FEL, FUBAR, and SLAC). The number of pervasive positive selection sites for NS1, NP1, NP, and VP1 was 11, 2, 11, and 9, respectively (Table 1). On the other hand, the number of episodic positive selection sites for NS1, NP1, NP, and VP1 were 27, 2, 9, and 50, respectively (Table S5). Sites under negative pressure for NS1, NP1, NP, and VP1 were 352, 36, 8, and 486, respectively (Table S5). Considering the length of the proteins, 15.94% of the codons presented pervasive positive selection for the NP gene. In the case of NS1, NP1, and VP1, the percentages of positive selection sites were 1.56%, 1.96%, and 1.33%, respectively.

### 3.5. Protein Modeling of Viral Capsid and T and B Epitope Prediction

The results of the modeling with AlphaFold2 produced 5 models for each protein analyzed (Figure S1, Table S6). In the case of VP1 of TuPV and ChPV, the overall quality of the models was High, with the maximum value of 77.58 of the mean predicted local distance difference test (pLDDT) for the best TuPV model and 74.62 for the best ChPV model. pLDDT values were High or Very High throughout VP1, except for the first 170 amino acids. In the case of VP2 of TuPV and ChPV, the overall quality of the models was High, with the maximum value of 82.21 of the mean predicted local distance difference test (pLDDT) for the best TuPV model and 82.59 for the best ChPV model. pLDDT values were High or Very High throughout VP2, except for the first 30 amino acids. Due to this, the three-dimensional representation in subsequent analyses was performed in reference to VP2.

**Figure 5:**
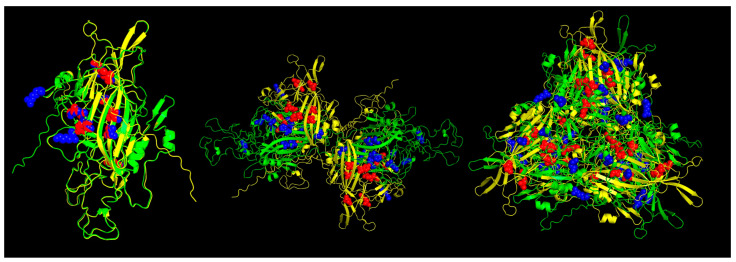
Predicted MHC-I strong binder epitopes from VP2 protein ChPV and TuPV. Protein alignment of monomers (**left**), dimers (**center**), and trimers (**right**). ChPV is colored green. TuPV is colored yellow. MHC-I epitopes for ChPV are colored blue. MHC-I epitopes for TuPV are colored red.

The results of the Pan-specific binding of peptides to MHC class I proteins using NetMHCpan-4.1 allowed for the identification of potential VP1 (and VP2) epitopes of 8-mer to 11-mer length and with characteristics of strong binders (defined as having % rank <0.5) (Table 2, Figure 5). The number of peptides predicted for ChPV was 16 and for TuPV was 15. Ten peptides correspond to the variants aligned at the same positions for ChPV and TuPV. The only conserved peptide for both was KEFFKNHQGA.

**Figure 6:**
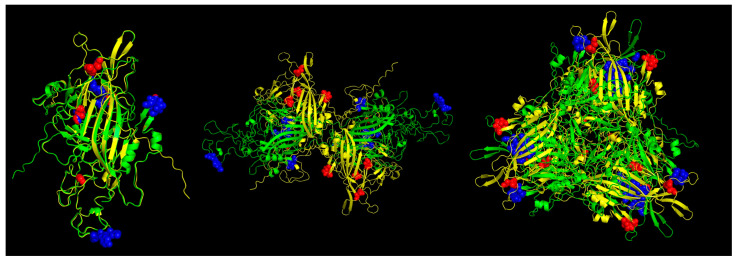
Predicted MHC-II strong binder epitopes from VP2 protein ChPV and TuPV. Protein alignment of monomers (**left**), dimers (**center**), and trimers (**right**). ChPV is colored green. TuPV is colored yellow. MHC-II epitopes for ChPV are colored blue. MHC-II epitopes for TuPV are colored red.

In the case of the Pan-specific binding of peptides to MHC class II proteins, the use of NetMHCIIpan-4.0 allowed for the identification of potential VP1 (and VP2) epitopes of 15-mer length with characteristics of strong binders (defined as having % rank <0.5) (Table 3, Figure 6). The number of predicted peptides for ChPV was 19 and for TuPV was 13. Nine peptides correspond to the variants aligned at the same positions for ChPV and TuPV. The only conserved peptide for both was RKRFFITQAQKNKKP.

## 4. Discussion

RSS is an enteric disease that causes significant economic losses in poultry farming. Despite multiple studies carried out, the etiological agent remains uncertain. The present retrospective study was carried out on samples of chickens affected with RSS using a viral metagenomic approach to explore de viral diversity and, consequently, the evolutionary characteristics on assembled Chicken Parvovirus genomes.

The outcomes of viral metagenomics revealed several viral families associated with diverse hosts. The occurrence of these organisms is likely linked to environmental factors, potentially introduced through sources like contaminated food or drink, or as components of the natural gut microbiota [19,37,38]. Conversely, the prevalence of parvovirus reads substantiated previously obtained results through molecular approaches [15]. Additionally, the presence of the avian leukosis virus was attributed to the endogenous (non-pathogenic) subtype of this virus. Lastly, since the genus *Aveparvovirus* within the family *Parvoviridae* exhibited a higher abundance, our focus was directed toward identifying the species within this genus. As a result, we assembled two near-complete genomes of Chicken Parvovirus (*Aveparvovirus galliform1*).

Our phylogenetic analysis for the major coding genes (NS1 and VP1) identified two main clades within *Aveparvovirus galliform1*. In both cases, Brazilian strains of this study clustered within the same branch, inside the ChPV clade, differentiating them from TuPV strains. Nucleotide identity analysis confirmed the same classification, with higher values against other strains from the ChPV clade. With this respect, the NS1 protein is pivotal for parvovirus pathogenicity [13,39]. Following its critical role, we have confirmed the minimal genetic variations among different ChPV strains as previously proposed [3,39], indicating significant functional conservation crucial for the virus’s stability. Conversely, the significant variability observed in VP1 underscores its pivotal role in facilitating adaptability [17,40]. Given these insights, the VP1 gene emerges as a compelling candidate for viral strain classification [3].

Genetic recombination plays a crucial role in the evolution and diversity of parvoviruses [41]. The exploratory recombination analysis allowed us to identify the most frequent breakpoint sites, highlighting four recombination hotspots, three of these in the NS1 gene and one in VP1. The incidence of recombination at these sites has been previously reported [17,39]. Then, with specific analyses, recombination events were identified in two regions of the USP-574-A strain. The first event was in the NS1 gene, where the major parent (MG602511) was related to China, and the associated RSS [17] was identified; the minor parent was related to USP-574-B from this study. Similarly, the second event occurred in the VP1 gene, with a major parent (KX133425) related to China and suspected of RSS [17], and the minor parent was related to USP-574-B of this study. The results suggest that the diversity gain in *Aveparvovirus* may be attributed to the frequent occurrence of recombination processes in its genes, as shown previously [17,39,42].

The analysis of selective pressure permits us to infer how evolutionary forces have influenced the extant genetic diversity from genomic sequencing data [32]. There are no previous studies of selective pressure in the genus *Aveparvovirus*; however, previous studies in other parvoviruses have described evolution under negative pressure in the main genes and a greater abundance of these sites when compared to sites under positive pressure [43,44,45,46,47]. Our analyses also revealed that most of the *Aveparvovirus galliform1* genome has evolved under purifying (negative) selection, except for NP, which presented proportionally more specific sites under positive selection. With this respect, NP is related to the efficient replication of the viral DNA and expression of capsid protein [48], and the incidence of positive selection could impact evolutionary adaptations.

The structural modeling of the viral capsid aligns with the classical architecture observed in single jelly-roll viruses [49,50]. Notably, the region attributed to VP2, which serves as the major capsid protein, demonstrated a higher degree of conservation, reflecting its fundamental role in capsid formation [4,6]. The comparative analysis of capsid proteins from ChPV and TuPV revealed a significant presence of strong T-cell epitopes, several of these on the same sites, underscoring the immunological significance of these sites. Despite co-localization, peptide sequences within these epitopes exhibited considerable diversity between ChPV and TuPV, with only a single shared epitope among all potential predicted T-cell epitopes. This immunological discrepancy may reflect and explain variations in host specificity. Additionally, most of these critical epitopes were distributed across the capsid, predominantly within the VP2 region, and several near the loops, consistent with previous reports of parvoviruses [51,52,53,54]. It is important to note that, to date, no specific TuPV or ChPV templates have been experimentally resolved or crystallized, which constitutes a limitation of the present study. Consequently, the modeling approach and predictions are approximations requiring subsequent experimental validation. Nonetheless, the comparison of potential T-cell epitopes between TuPV and ChPV underscores potential differences in the immune response to both viruses.

The results of the present study allow us to show the incidence and significance of ChPV in the pathogenic virome in birds with RSS. However, the causal association between ChPV and RSS in the present study cannot be established in the absence of experimental infection studies. Furthermore, the coexistence of different strains of *A. galliform1* or other related parvoviruses may allow diversification through evolutionary mechanisms such as genomic recombination and selective pressure, as is common in parvoviruses and other avian viruses [17,42,47,54,55,56,57]. Although the majority of parvoviruses in diverse environments evolve largely under purifying forces of selection (negative selection), it is important to identify potential antigenic hot spots that more dramatically alter the effects on evolution, diversification and distancing (ChPV and TuPV as an example), the determination of host and tissue tropism, modulation and immunological escape, and the subsequent influence on the pathogenic characteristics as has been reported in other cases of viral infections [3,17,40,46,50,52,58,59,60].

## Figures and Tables

**Table 1:** Sites under pervasive positive selection pressure in the *Aveparvovirus galliform1* coding genes.

Gene	Codon Position	FEL *p*-Value ^2^	FUBAR Probability α < β Positive ^2^	SLAC P-[dN/dS > 1] ^2^
NS1	131	—	0.006	
	331	—	0.042	—
	349	—	0.020	—
	534	0.0819	0.015	—
	615	0.0182	0.006	0.0879
	617 ^1^	0.0940	—	—
	637	0.0098	0.000	0.0223
	640	0.0018	0.000	0.0061
	645	0.0044	0.000	0.0303
	647	0.0003	0.000	0.0018
	654	0.0133	0.006	0.0403
NP1	2	0.0872	0.934	—
	6	0.0855	0.946	—
NP	6	0.0133	0.972	—
	15	0.0737	0.956	—
	16	—	0.948	—
	31	—	0.904	—
	35	0.0751	—	—
	45	—	0.917	—
	58	0.0835	0.916	—
	66	—	0.930	—
	67	0.0822	—	—
	68	0.0887	—	—
	69	0.0045	0.995	0.0427
VP1	8	0.0360	0.918	0.0702
	85	0.0777	—	—
	107	0.0342	0.904	0.0517
	134	0.0724	0.918	—
	187	0.0042	0.983	—
	188	0.0416	0.916	0.0677
	192	0.0261	0.940	0.0618
	210	0.0657	—	—
	215	0.0056	0.983	0.0214

^1^ Detected only in TuPV. ^2^—: no signal detected.

**Table 2:** MHC-I strong binder epitopes from VP1 (and VP2) protein predicted for *Aveparvovirus galliform1*.

VP1 Position ^1^	Peptide		Chicken MHC-I Substitute Alleles ^2^		
	ChPV	TuPV	HLA-B*40:06	HLA-B*41:03	HLA-B*41:04
47	ARKELTPQQKA	—	C	—	—
48	RKELTPQQKA	KKELTAQQKA	C + T	—	—
49	KELTPQQKA	KELTAQQKA	C + T	C + T	C
84	KEFFKNHQGA	KEFFKNHQGA	C + T	—	—
128	EEHPFNQEEL	EEAPFNEQEL	—	C + T	—
135	—	NEQELEEAM	—	T	—
202	RDMDQYKAI	RDFDKYQAI	T	C + T	C + T
221	SENQTQYF	AENETQYF	—	C + T	C + T
221	—	AENETQYFGF	—	T	T
301	QEGKYPRL	—	—	—	C
301	QEGKYPRLL	QEGRYPRIL	—	C + T	C + T
353	RESAFYCL	—	—	C	C
372	NEWETTFVF	NEWQTSYEF	C + T	C + T	C + T
378	—	YEFPDSTP	T	—	—
446	LENLANVAV	—	C	C	C
469	RPESDKDEYL	RPETDKDEYL	—	C + T	—
581	KESPGHIF	KESPGHVF	—	C + T	C + T
581	KESPGHIFV	KESPGHVFV	C + T	C + T	C + T
613	VEIEWELEP	—	C	—	—
615	—	IEWELEHFT	T	—	—
Total peptides			16	22	17

^1^ VP2 starts at position 143; ^2^—: not binding. C: ChPV. T: TuPV.

**Table 3:** MHC-II strong binder epitopes from VP1 (and VP2) protein predicted for *Aveparvovirus galliform1*.

VP1 Position ^1^	Peptide		Chicken MHC-II Substitute Alleles ^2^			
	ChPV	TuPV	DRB1:1310	DRB1:1366	DRB1:1445	DRB1:1482
5	APKGYVPSLPTTDEE	PPKGYVPSLPTTDEE	C + T	—	—	—
6	PKGYVPSLPTTDEEA	—	C	C	—	—
58	ERKRFFITQAQKNKK	DRKRFFITQAQKNKK	C + T	—	—	—
59	RKRFFITQAQKNKKP	RKRFFITQAQKNKKP	C + T	C + T	—	—
293	GTIQIFADQEGKYPR	GTIQIFADQEGRYPR	—	—	—	C + T
295	—	TIQIFADQEGRYPRI	—	—	—	T
403	LYDTWNVNGRGDDAK	—	C	C	—	—
404	YDTWNVNGRGDDAKR	—	C	C	—	—
405	DTWNVNGRGDDAKRG	—	C	C	—	—
431	—	GPYIYLSDTTAAGQQ	—	T	—	—
494	VRNSQIQVSTANKVQ	—	—	—	C	C
495	RNSQIQVSTANKVQV	—	—	—	C	C
496	NSQIQVSTANKVQVD	—	—	—	C	C
497	SQIQVSTANKVQVDT	—	—	—	C	C
498	QIQVSTANKVQVDTS	—	—	—	C	C
585	GHIFVKVTPKPTGAA	GHVFVKVTPKPTGAA	—	—	C + T	—
586	HIFVKVTPKPTGAAN	HVFVKVTPKPTGAAN	—	—	C + T	—
648	DENGQYQVNVNSGDI	DENGQYQINTTSADL	C + T	T	—	—
649	ENGQYQVNVNSGDIT	ENGQYQINTTSADLA	C + T	C + T	—	—
650	NGQYQVNVNSGDITR	NGQYQINTTSADLAR	C + T	C + T	—	—
651	—	GQYQINTTSADLARL	T	T	—	—
661	DITRLYMTKRAPRTN	—	—	—	C	—
Total peptides			17	13	10	8

^1^ VP2 starts at position 143. ^2^ —: not binding. C: ChPV. T: TuPV.

## Data Availability

Two near-complete *Aveparvovirus galliform1* genomes were assembled from the filtered reads and deposited in GenBank with accession numbers PP329606 (USP-574-A) and PP329607 (USP-574-B).

## Supplementary Materials

The following supporting information can be downloaded at https://www.mdpi.com/article/10.3390/v16091389/s1. Table S1. Assembled and filtered reads only corresponding to viruses; Table S2. Identity matrix of nucleotide sequences from the complete NS1 sequences of ChPV strains used in this study; Table S3. Identity matrix of nucleotide sequences from the complete VP1 sequences of ChPV strains used in this study; Table S4. Recombination events of the complete coding genomes of *Aveparvovirus galliform1* strains; Table S5. Selection pressure results in the coding genomes of *Aveparvovirus galliform1*; Table S6. Results of protein modeling with AlphaFold2 for VP1 and VP2 of TuPV and ChPV. Figure S1. AlphaFold3 prediction metrics of viral capsid proteins of *Aveparvovirus*. (Left Side) Predicted Local Distance Difference Test (pLDDT) and (Right Side) MSA Sequence Coverage of A) VP1 of TuPV; B) VP1 of ChPV; C) VP2 of TuPV; D) VP2 of ChPV.
