# Supplementary material for: Molecular and Evolutionary Characteristics of Chicken Parvovirus (ChPV) Genomes Detected in Chickens with Runting–Stunting Syndrome
Source: Viruses. 2024 Aug 30;16(9):1389. doi: 10.3390/v16091389 (PMC11436221; doi:10.3390/v16091389)
Supplement: Supplementary file 1 [file viruses-16-01389-s001.zip › viruses-3078631-supplementary figure S1.pdf]

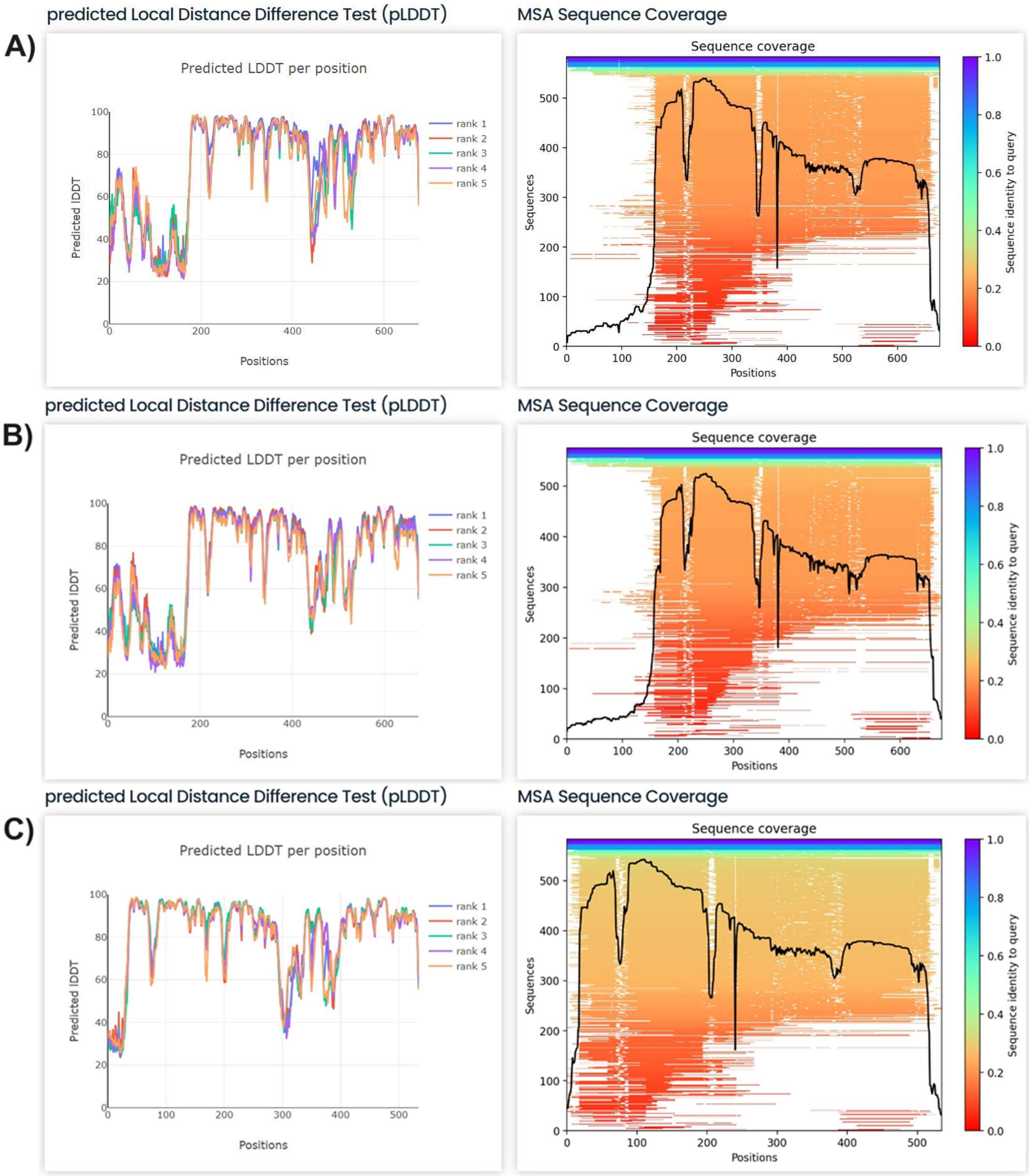
A)
B)
C)
predicted Local Distance Difference Test (pLDDT)
MSA Sequence Coverage
Predicted LDDT per position
Predicted lDDT
Positions
rank 1
rank 2
rank 3
rank 4
rank 5
Sequence coverage
Sequences
Sequence identity to query

D)

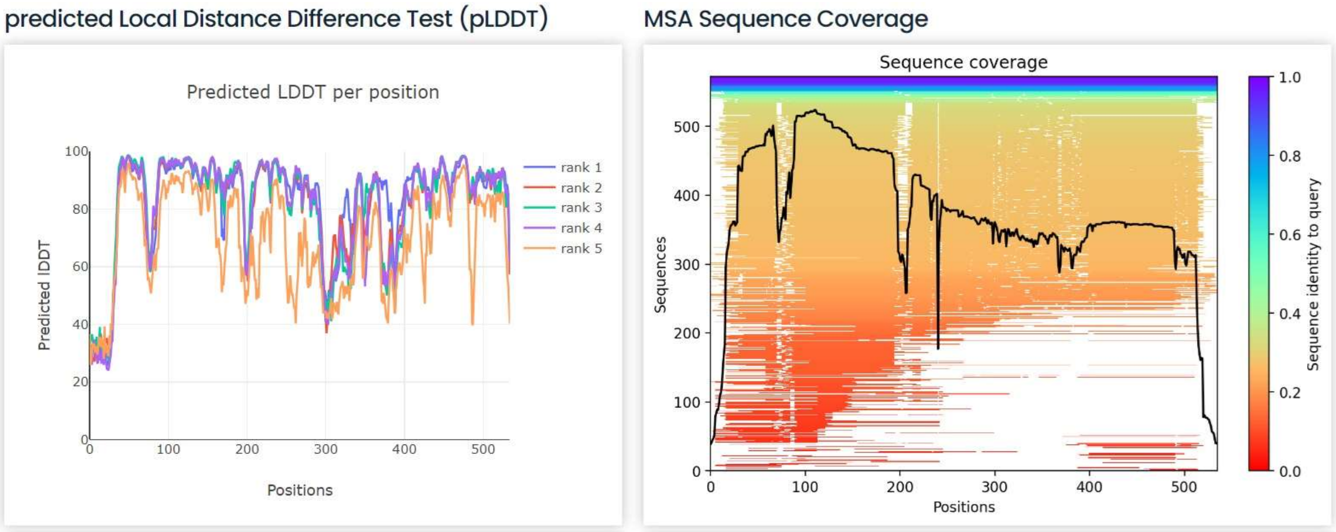

Figure S1. AlphaFold3 prediction metrics of viral capsid proteins of *Aveparvovirus*. (Left Side) Predicted Local Distance Difference Test (pLDDT) and (Right Side) MSA Sequence Coverage of: **A**) VP1 of TuPV; **B**) VP1 of ChPV; **C**) VP2 of TuPV; **D**) VP2 of ChPV.
